# Gut microbiota modulation: a key determinant of atopic dermatitis susceptibility in children

**DOI:** 10.3389/fmicb.2025.1549895

**Published:** 2025-04-28

**Authors:** Huimiao Tang, Wenxin Li, Yidan Xu, Yanjun Zhou, Michael R. Hamblin, Xiang Wen

**Affiliations:** ^1^Department of Dermatology, West China Hospital, Sichuan University, Chengdu, China; ^2^Laboratory of Dermatology, Clinical Institute of Inflammation and Immunology, Frontiers Science Center for Disease-related Molecular Network, West China Hospital, Sichuan University, Chengdu, China; ^3^The International Department of Chengdu Shude High School, Chengdu, China; ^4^Laser Research Centre, Faculty of Health Science, University of Johannesburg, Doornfontein, South Africa

**Keywords:** atopic dermatitis, children, gut microbiome, structural feature, influencing factor

## Abstract

Atopic dermatitis is a chronic inflammatory skin condition with a higher incidence rate among children. In recent years, the role of the gut microbiota in the pathogenesis of atopic dermatitis has garnered increasing attention. This review systematically delineates the research advancements in the structural characteristics of the gut microbiota in children with atopic dermatitis and its influencing factors. Studies have revealed significant differences in the gut microbiota structure between children with atopic dermatitis and healthy controls, characterized by a reduction in microbial diversity, a decrease in beneficial bacteria, and an increase in harmful bacteria. Dietary patterns, environmental factors, birth patterns, antibiotic use, and gestational diabetes mellitus are factors could impact the gut microbiota hence influencing the susceptibility of children to atopic dermatitis. Moreover, this review explores the interplay between the gut microbiota and the immune system in atopic dermatitis, with the potential to inform more effective probiotic treatment strategies for children with atopic dermatitis.

## 1 Introduction

Atopic dermatitis (AD) is a prevalent chronic inflammatory dermatosis characterized by pruritic, dry skin and the recurrence of symmetric eczematous eruptions, AD patients also show elevated serum IgE levels and can have allergic rhinitis/conjunctivitis or asthma either themselves or in close relatives (Saeki et al., 2022). The incidence of AD is on the rise globally, particularly among children, the prevalence of AD in children has gradually increased up to 20%, severely impairing the quality of life of patients and imposing a significant burden on public health systems (Laughter et al., 2021). Despite extensive research endeavors to explore the etiology and pathogenesis of AD, its complex pathological processes remain not entirely elucidated (Li et al., 2021; Kantor and Silverberg, 2017).

The pathogenesis of AD encompasses genetic predisposition, skin barrier dysfunction, immune dysregulation, and environmental factors. Key genetic factors include loss-of-function variants in the filaggrin (FLG) gene, which compromise the integrity of the skin barrier (Schuler et al., 2024). The immune response is significantly influenced by T helper (Th) cell subsets like Th1 cells, Th2 cells, and Th17 cells, which produce cytokines such as IL-4, IL-13, and IL-31, thereby activating inflammatory pathways and fostering the progression of AD. Mast cells and basophils contribute to inflammation and pruritus through the release of IL-13 and leukotrienes, respectively (Schuler et al., 2024).

With the rapid advancement of microbiomics, in recent years, the role of the gut microbiota in maintaining human health and disease pathogenesis has garnered widespread attention. Studies has indicated that an imbalance in the gut microbiota was associated with the onset and progression of numerous skin diseases including AD (De Pessemier et al., 2021). The intestinal microbiome, by modulating the function and activity of immune cells, influences the homeostasis of the skin immune system. Its metabolic products, such as short-chain fatty acids (SCFAs), can impact skin health through circulation. Additionally, the microbiome can also affect the generation and release of neurotransmitters and hormones, thereby influencing the neurovascular regulation and immune function of the skin (De Pessemier et al., 2021). Childhood is a critical period for gut microbiota colonization and maturation (Rodríguez et al., 2015). Therefore, investigating the structural characteristics of the gut microbiota in children with AD and the influencing factors is of great significance for understanding the disease’s pathogenesis.

However, current research on the gut microbiota of pediatric AD yields inconsistent findings, and there is a lack of systematic analysis regarding the specific factors affecting the structure of the gut microbiota. This review aims to systematically synthesize the research progress on the structural features of the gut microbiota in pediatric AD and its interaction with the immune system, and to investigate how factors such as diet, environment, mode of delivery, antibiotic usage, and gestational diabetes mellitus (GDM) may modulate the susceptibility to pediatric AD by acting upon the gut microbiota. Through these discussions, we intend to provide novel perspectives for the prevention and treatment of AD.

## 2 Gut microbiome in pediatric AD

The gut microbiota interacts with the host immune system, participating in the regulation of innate, adaptive, and autoimmunity, thereby influencing the overall health and disease susceptibility of the host. They play a vital role in the normal processes of digestion and absorption of food. Breast milk and formula milk are the primary sources for the establishment of gut microbiota colonization and the supplementation of SCFAs in early childhood (Davis et al., 2022). After moving from the gut to the skin through the circulation, SCFAs and metabolites can modulate the immune response of the skin, decreasing the production of inflammatory mediators, and maintaining host immune competence (Yao et al., 2022). Study has shown that Gut microbiota cells affect metabolic pathways involving tryptophan, which play a significant role in host immunity. The aryl hydrocarbon receptor (AhR), a ligand-activated transcription factor involved in diverse physiological processes including immune regulation, can be activated by indole derivatives such as indole-3-lactic acid (ILA), indole-3-carbaldehyde (I3C), and indole-3-acetic acid (IAA) produced by *Bifidobacterium longum* through tryptophan metabolism, thereby mediating immune responses (Fang et al., 2022). ILA is the main metabolite of *Bifidobacterium* in the intestinal tract of infants, which can react with downstream factors to reduce the production of TNF-*α* and lipopolysaccharide (LPS), and reduce the inflammatory response related to IL-8 in epithelial cells (Ehrlich et al., 2020). I3C can inhibit the production of thymic stromal lymphopoietin (TSLP), and reduce Th2 immune responses, thereby alleviating the symptoms of AD (Fang et al., 2022). Additionally, *B. longum* can also increase the *β*-diversity of the gut microbiota (Fang et al., 2022).

*Bacteroides fragilis* favors the production of butyrate and propionate in the gut, infants with allergen-sensitized atopic eczema at 3 weeks of age were characterized by enrichment of *Escherichia coli* and *Klebsiella pneumoniae*, which produce virulence factors such as invagins, adectin, flagellin, and lipopolysaccharide, results in delayed colonization of *bacteroides* and decreased content of butyrate and propionate (Ta et al., 2020). Park et al. found that in 6-month-old infant with mild AD, the relative abundance of *Streptococcus* was positively correlated with the severity of AD, while that of *Clostridium* was negatively correlated, infants with transient AD had lower levels of butyrate and valerate than healthy and persistent AD infants (Park et al., 2020). However, in another study, Yu et al. found that the prevalence of *Streptococcus* was lower in infants with AD (Yu et al., 2021). Previously, Penders et al. collected fecal samples from 957 participating infants at 1 month of age and conducted home visits at 2 years, they discovered that early colonization with *Escherichia coli* and *Clostridium difficile* could exacerbate the risk of eczema in infants and proposed that atopic symptoms may arise due to the crowding out of beneficial microbes by these two species, leading to dysregulated induction of regulatory T cells, insufficient transformation of regulatory T cells is the main cause of Th1 and Th2 immune imbalance, thus increasing susceptibility to AD (Penders et al., 2007). In a study by Wopereis et al. employed pyrosequencing of fecal samples collected from infants at 4 and 26 weeks postpartum, it was found that compared to healthy controls, children with infantile eczema within the first 18 months after birth had a reduced richness of *Bifidobacterium* in the gut at 26 weeks postpartum, they also showed increased abundance of gut *Parabacteroides* and *Enterobacteriaceae*, along with decreased populations of *Eubacterium* (butyrate producing)*Clostridium*, *Lachnospiraceae* and anaerobes (Wopereis et al., 2018).

The gut microbiota in children is dynamic and evolves over time, gradually tending to resemble that of adults. Pediatric AD often exhibit an ecological imbalance in the gut microbiota (Kumbhare et al., 2019). However, the heterogeneity of the imbalanced patterns of gut microbiota observed in the aforementioned results is significant. Variables such as the different age strata of the study participants, climatic conditions across various regions, different ways of feeding, the prenatal maternal pregnancy status, mode of delivery, and the presence or absence of older siblings may all potentially influence the outcomes. These are areas that this review seeks to delve into further.

## 3 Influencing factors of gut microbiota in children

### 3.1 Diet

The neonatal gut microbiota is not yet fully established. Therefore, breastfeeding provides an array of health-promoting microorganisms and essential nutrients that enhance the diversity of the infant intestinal microbiota (Pantazi et al., 2023). Human milk oligosaccharides (HMOs) are crucial factors in the early development of the infant gut microbiota. They serve as prebiotics that are not digested by the infant, but can still be utilized by beneficial gut bacteria, thereby promoting the growth and proliferation of these microorganisms (Rahman et al., 2023). HMOs specifically facilitate the colonization of *Bifidobacteria* and *Lactobacilli* in the infant intestine, which aid in food digestion, and produce SCFAs and ILA. These compounds can be further absorbed by the intestinal epithelium and contribute to the establishment and maintenance of the infant gut immune function (Davis et al., 2022).

Additionally, breastfeeding protects the infant gut by transferring maternal antibodies, such as IgA, IgG, and cytokines, which can effectively resist harmful microorganisms and viruses in the gut. Breastfeeding promotes the maturation of the intestinal mucosal immune system and improves the barrier function of the gut (Davis et al., 2022). It has been shown that prolonged breastfeeding increases the abundance of *Veillonella, Rothia, Streptococci, Bifidobacteria, Haemophilus,* and *Staphylococci* in the infant gut. *Rothia, Veillonella,* and *Bifidobacteria* play important roles in maintaining intestinal homeostasis, while *Veillonella* can convert lactate to propionate and butyrate (Fehr et al., 2020). At 6 months of age, the dominant gut microbiota of infants can vary based on diet, with *Bifidobacterium* being the predominant genus in breastfed infants, while *Escherichia/Veillonella* is dominant in those receiving mixed feeding (Lee et al., 2018). Infants weaned from breast milk to formula exhibit a more adult-like microbiota, characterized by the presence of *Roseburia, Clostridium,* and *Anaerostipes* (Bäckhed et al., 2015). Table 1 presents a series of clinical studies investigating the factors influencing the gut microbiota in children. One study by Odamaki et al. demonstrated that the relative abundance of *Actinobacteria* was significantly lower after weaning, and was further reduced with increasing age. *Firmicutes* became the predominant phylum in the post-weaning gut microbiota of children, with higher counts observed in children over 4 years old (Odamaki et al., 2016). In a systematic analysis, it was suggested that oral administration of probiotics to pregnant and lactating mothers was more effective in preventing the onset of AD in children before the age of 2, compared to direct infant supplementation (Husein-ElAhmed and Steinhoff, 2023).

**Table 1:** Summary of clinical studies on gut microbiota changes under different conditions in children.

Study type	Year	Country	Sample Size	Age	Study Focus	Grouping	Enriched microbial communities
Case–control study	2018 (Lee et al., 2018)	Korea	63	6 months	Feeding	Breast-fed	*Bifidobacterium*
						Mixed-fed	*Escherichia, Veillonella*
Clinical study	2010 (Dominguez-Bello et al., 2010)	America	10	<1 day	Birth pattern	Vaginal delivery	*Lactobacillus, Prevotella, Bacteroides*
						Cesarean section	*Staphylococcus, Corynebacterium, Propionibacterium*
Cross-sectional study	2021 (Seppo et al., 2021)	America	104	2 months	Environment	Rural	*Bifidobacteriaceae, Clostridiaceae, Aerococcaceae*
						Urban	*Enterobacteriaceae*
Cohort study	2006 (Penders et al., 2006)	Netherlands	1,032	1 month	Birth pattern	Cesarean section	*C difficile*
						Vaginal delivery	*Bifidobacteria, Bacteroides*
						No-first-born	*Bifidobacterium*
					Feeding	Breast-fed	*Bifidobacterium*
						Formula-fed	*E. coli, Clostridium difficile, Bacteroides fragilis, Lactobacillus*
					Antibiotics	Antibiotics usage	*Bifidobacterium, Bacteroides fragilis*
Cohort study	2020 (Soderborg et al., 2020)	America	46	2 weeks	Gestational diabetes mellitus (GDM)	Increased in GDM	*Phascolarctobacterium, Lachnospiraceae*
						Decreased in GDM	*Lactobacillus, Flavonifractor, Lactobacillaceae, Rikenellaceae, Erysipelotrichaceae*
Cohort study	2020 (Crusell et al., 2020)	Denmark	125	1 week-9 months	GDM	Increased in GDM	*Isobaculum, Carnobacteriaceae, Turicibacter, Faecalibacterium, Clostridium XVIII*
Longitudinal Study	2020 (Galazzo et al., 2020)	Netherlands	440	5-31 weeks	Birth pattern	Vaginal delivery	*Bacteroides*
						Cesarean section	*Klebsiella, Citrobacter, Leclercia, Raoultella*
					Feeding	Breast-fed	*Bifidobacteria, Staphylococci, Streptococci*
						Formula-fed	*Pseudobutyrivibrio, Lachnobacterium, Roseburia, Blautia*
Longitudinal cohort study	2015 (Bäckhed et al., 2015)	Sweden	98	first year of life	Feeding	Formula-fed	*Bacteroides, Bilophila, Roseburia, Clostridium, Anaerostipes*
						Breast-fed	*Bifidobacterium, Lactobacillus, Collinsella, Megasphaera, Veillonella*
					Birth pattern	Vaginal delivery	*Bacteroides, Bifidobacterium, Parabacteroides, Escherichia, Shigella*
						Cesarean section	*Enterobacter hormaechei, E. cancerogenus, Haemophilus parainfluenzae, H. aegyptius, H. influenzae, H. haemolyticus, Staphylococcus saprophyticus, S. lugdunensis, S. aureus, Streptococcus australis, Veillonella dispar*
Longitudinal Study	2013 (Penders et al., 2013)	Germany	606	5-31 weeks	Birth pattern	Cesarean section	*Clostridium cluster I, C difficile, Clostridia*
						Vaginal delivery	*Bifidobacteria, Bacteroides, Lactobacilli*
						No-first-born	*Lactobacilli, Bacteroides*
					Feeding	Breast-fed	*Lactobacilli, Bifidobacteria*

However, certain compounds from breast milk, such as long-chain saturated fatty acids, may elicit a type 3 innate lymphoid cell-mediated inflammatory response in the infant gut, which have been observed to migrate from the gut to the skin in mice. This could increase the incidence of AD in infants, which may be associated with unhealthy lifestyle and dietary choices of the mother (Kong et al., 2021). A recent study found that the concentration of arachidonic acid in breast milk tends to increase gradually with the prolongation of lactation, which may lead to an imbalance in the infant’s gut microbiota. In infants with AD, this imbalance is likely to facilitate the conversion of arachidonic acid into allergenic and pro-inflammatory substances, such as prostaglandins and leukotrienes, while also elevating the levels of IgE in peripheral blood, thereby exacerbating the symptoms of AD. This research contributes to the understanding of the etiology of AD in infants prior to the introduction of complementary foods (Jiang et al., 2024).

Long-term consumption of unprocessed milk has been found to increase the richness of the gut microbiota in 1-year-old infants (Pechlivanis et al., 2023). Host serum vitamin D levels can regulate the composition of the gut microbiota through the gut-skin axis, leading to increases in *α* and *β* diversity of gut microbes such as *Lachnospira* and *Fusicatenibacter* (Bosman et al., 2019). A systematic review including 32 randomized controlled trials showed that oral vitamin D supplementation significantly reduced the severity of skin symptoms in AD patients compared to a control group. Although the specific mechanism requires further investigation, it may be related to reduced colonization by *Staphylococcus aureus* on the skin (Li et al., 2022).

### 3.2 Environment

The hygiene hypothesis proposes that the reduced microbial exposure people receive in urbanized, hygienic environments leads to an increase in Th2-mediated allergic responses (Yazdanbakhsh et al., 2002). It had been shown that exposure to soil, indoor dust, and decayed plants can enhance the richness and diversity of gut microbiota, thereby reducing serum total IgE levels and enhancing host immune function (Zhou et al., 2016). Early-life exposure to farm animals has been associated with protection against pollen sensitization in children, possibly due to increased gut microbiota richness (Pechlivanis et al., 2023). Infants who were traditionally raised in large farm families have been found to have a lower risk of atopic diseases, and their fecal samples were rich in *Bifidobacteriaceae, Clostridiaceae,* and *Aerococcaceae* (Seppo et al., 2021). Exposure to unpasteurized milk on farms also increases immunity in infants, leading to higher levels of IFNγ and TNFα, and promoting the function of Tregs to limit allergic responses (Jackson et al., 2022). Furthermore, a study showed that there were differences in the microbial composition of dust between farm and urban environments, with rural environments having more *Ruminococcaceae, Lachnospiraceae,* and *Bacteroidaceae families* compared to cities. Inhaled indoor dust may facilitate the colonization of microbes in the gut. In farm environments, the relative abundance of *Clostridiales, Ruminococcaceae,* and *Bacteroidales* in AD children was significantly lower compared to healthy children, whereas these differences were not significant in urban environments, possibly due to a stronger immune response to environmental microbes in rural children (Mahdavinia et al., 2021).

### 3.3 Birth pattern

Studies have indicated that infants born by Cesarean section (CS) have a higher risk of developing atopic diseases and asthma, and also show a reduction in gut microbiota diversity (Sandall et al., 2018). Infants born by vaginal delivery (VD) show a microbiota composition that is more similar to that of their mothers (Bäckhed et al., 2015). Table 2 illustrates the differences in gut microbiota colonization between VD and CS as observed in various studies. The gut microbiota of VD-born infants are communities of *Lactobacillus, Prevotella,* or *Bacteroides,* which are similar to those found in the mother’s vagina, and are thought to be passed from mother to child. On the other hand, the microbiota of CS-born infants is dominated by *Staphylococcus, Corynebacterium,* and *Propionibacterium*, resembling the bacterial communities found on the mother’s skin (Dominguez-Bello et al., 2010).

**Table 2:** Difference of gut microbiota colonization between vaginal delivery (VD) and cesarean section (CS).

Detection method	Country	No. of VD	Gut microbiota of VD	No. of CS	Gut microbiota of CS	Reference
Shotgun-sequenced stool	Sweden	83	*Bacteroides, Bifidobacterium, Parabacteroides, Escherichia, Shigella*	15	*Enterobacter hormaechei, Enterobacter cancerogenus, Haemophilus parainfluenzae, Haemophilus aegyptius, Haemophilus influenzae, Haemophilus haemolyticus, Staphylococcus saprophyticus, Staphylococcus lugdunensis, Staphylococcus aureus, Streptococcus australis, Veillonella dispar, Veillonella parvula*	Bäckhed et al. (2015)
Multiplexed 16S rRNA gene pyrosequencing	America	4	*Lactobacillus, Prevotella, Sneathia* spp.	6	*Staphylococcus, Corynebacterium, Propionibacterium* spp	Dominguez-Bello et al. (2010)
16S rDNA gene sequencing	Germany	378	*Bifidobacteria, Bacteroides, Lactobacilli*	119	*Clostridium cluster I, Clostridium difficile*	Penders et al. (2013)
16S rDNA gene sequencing	Netherlands	902	*Bifidobacteria, Bacteroides, Bacteroides fragilis*	118	*Clostridium difficile*	Penders et al. (2006)
16S rRNA gene sequencing	China	37	*No significant difference*	25	No significant difference	Liu et al. (2024)

A systematic review including 9 studies found that children born by CS had a higher probability of developing eczema before the age of 1 year compared to those born vaginally (Xiong et al., 2022). Galazzo et al. found that the abundance of the gut microbial genus *Bacteroides* was higher in VD-born infants at 5 to 31 weeks of age, compared to CS-born infants (Galazzo et al., 2020). Penders et al. reported that CS-born infants were more prone to colonization by *Clostridium difficile* and had higher counts compared to infants born normally. Their fecal microbiota contained fewer *Bifidobacterium, Bacteroides,* and *Escherichia coli*, while by 31 weeks of age, the dominant gut microbiota in naturally born infants shifted to *Lactobacillus* (Penders et al., 2013). This finding was in agreement with the findings of previous studies by Bäckhed et al. (2015) and Penders et al. (2006). However, recently Liu et al. reported that there was no significant differences in gut microbiota richness between infants with AD born by different delivery methods (Liu et al., 2024). A Greek cohort study suggested that CS did not significantly increase the risk of AD in children (Stefanaki et al., 2023). Zachariassen et al. carried out a cohort study suggesting that human CS delivery and the characteristic gut microbiota transmitted by CS did not increase the risk of AD in infants at 1 month of age. These researchers also transplanted gut microbiota from human infants delivered by either CS or VD into BALB/c mice, and found that the gut microbiota transmitted from CS infants elicited a higher immune response in oxazolone-induced dermatitis mice. These mice had higher serum IgE levels, and contained more Th2 cells in the auricular lymph nodes than the mice receiving VD-transmitted microbiota; however, there was no significant increase in the production of Th2-related cytokines (Zachariassen et al., 2023).

In addition to the mode of delivery, the birth order can also affect the establishment of the infant gut microbiota. The number of *Bifidobacterium* was higher in infants with siblings (Penders et al., 2006). Compared to the first-born child, infants with older siblings had higher levels of *Lactobacillus* and *Bacteroides* colonization and lower levels of *Clostridium,* from the fifth week of life to 31 weeks, potentially reducing the risk of AD (Penders et al., 2013).

### 3.4 Use of antibiotics

There is ample evidence to support the notion that maternal exposure to antibiotics during pregnancy is associated with an increased risk of AD in the offspring (Wan and Yang, 2023; Mubanga et al., 2021; Chiesa Fuxench et al., 2023). Research has also found that antibiotic use within the first year of life was the most important factor in increasing the risk of AD, with no significant differences observed between the use of broad-spectrum and narrow-spectrum antibiotics (Mubanga et al., 2021). In one systematic review, infants prenatally exposed to maternal antibiotics were found to have a relative decrease in the abundance of *Bifidobacterium* and *Bacteroidetes*, while the relative abundance of *Firmicutes* and *Proteobacteria* was higher (Grech et al., 2021). This finding was consistent with previous results which demonstrated a dose-dependent effect (Milliken et al., 2019). A cohort study containing 2,453 mother–child pairs found that the timing of antibiotic use during pregnancy significantly affected the development of AD in the offspring, with sulfamethazine being the main antibiotic associated with an AD risk in the first trimester and ciprofloxacin in the second trimester (Geng et al., 2021). Panduru et al. identified the use of antibiotics in the last 3 months of pregnancy as the most significant factor associated with AD in a cross-sectional study containing 1,046 subjects (Panduru et al., 2020). Early postnatal use of broad-spectrum antibiotics can lead to an imbalance in the infant gut microbiota, resulting in a decrease in key probiotics such as *Bifidobacterium* and *Bacteroides*, metabolic disorders, and an increased susceptibility to pathogen invasion (Penders et al., 2006; Vangay et al., 2015).

### 3.5 Gestational diabetes mellitus

The dynamics of gut microbiota during gestation and early infancy have attracted increasing research attention. Disruptions in microbial colonization patterns have been linked to subsequent development of inflammatory conditions, allergic diseases, and metabolic dysfunction of the immune system (Sokou et al., 2024). Gestational diabetes mellitus (GDM) is considered to alter the composition of the intestinal microbiota in newborns, reducing the richness of the gut microbiota, which primarily includes a decrease in the *Lactobacillaceae* in two-week-old infants, and can also affect the gut microbiota that potentially participate in the innate immune system function of newborns (Soderborg et al., 2020). Crusell et al. have found that neonates aged 1 week born to mothers with GDM exhibit lower richness of gut microbiota compared to those born to healthy mothers, but this difference becomes less pronounced by the time the neonates reach 9 months of age (Crusell et al., 2020). This change may be attributed to vertical transmission, which results in similar compositions of gut microbiota between GDM mothers and their offspring, thereby influencing metabolism. The abundance of *Firmicutes, Proteobacteria,* and the anti-inflammatory bacterium *Pharscolarctobacterium* is reduced in GDM neonates, *Prevotella copri* abundance is also decreased in GDM descendants at both birth and 9 months of age, conversely, one species of *Staphylococcus* is enriched in GDM neonates (Crusell et al., 2020).

**Figure 1:**
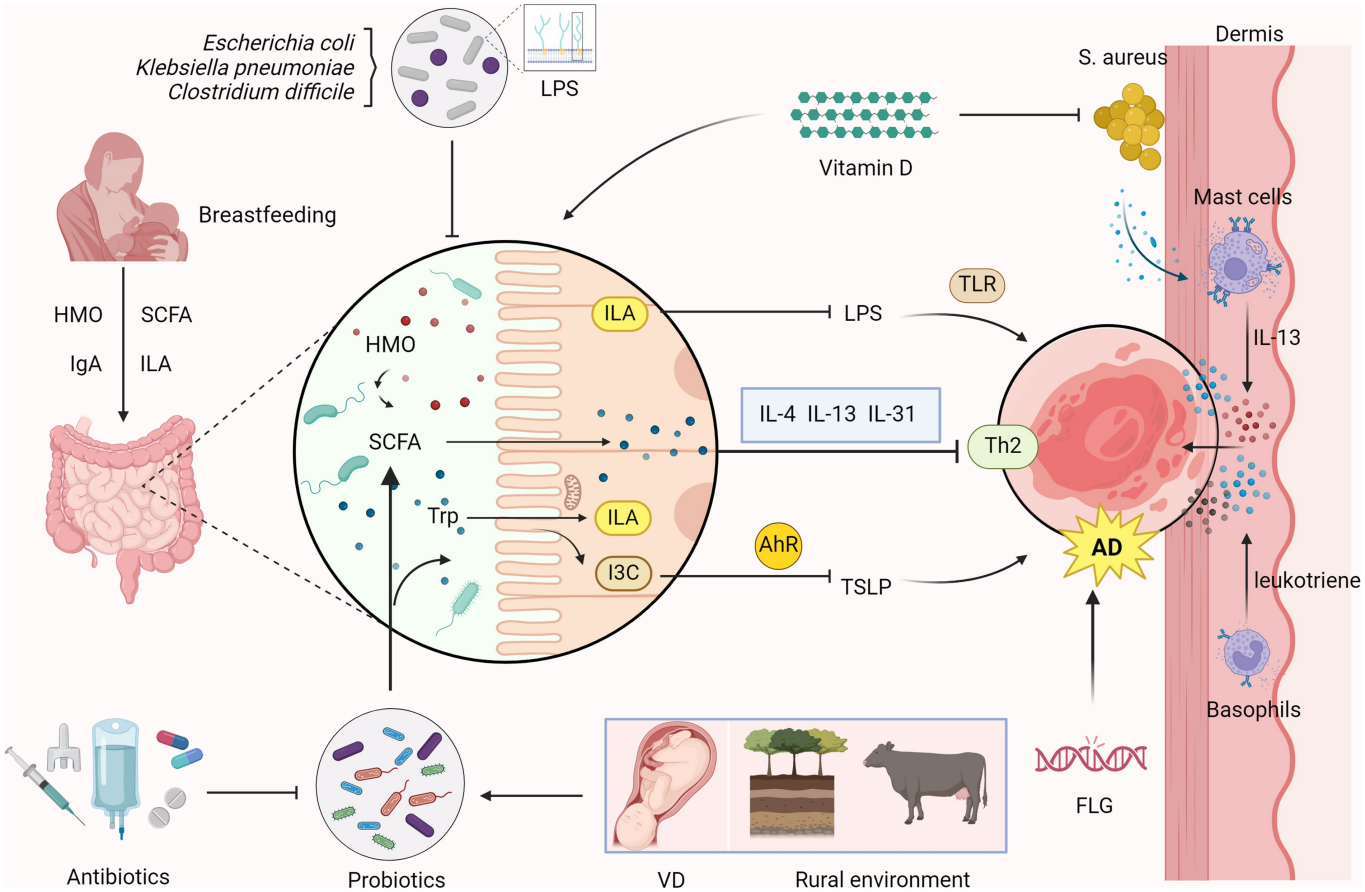
Relationship between intestinal microecology and AD in children Overgrowth of microorganisms such as *Escherichia coli, Klebsiella pneumoniae*, and *Clostridium difficile* perturbs gut microbiota homeostasis. Probiotics stimulate short-chain fatty acids (SCFAs) synthesis and generate indole-3-lactic acid (ILA) and Indole-3-carbaldehyde (I3C) through the metabolism of tryptophan (Trp). ILA inhibits the production of lipopolysaccharide (LPS), reducing toll-like receptor (TLR)-mediated inflammatory responses. I3C suppresses the production of thymic stromal lymphopoietin (TSLP) via the aryl hydrocarbon receptor (AhR)-mediated immune response, which reduces Th2 immune responses, alleviating atopic dermatitis (AD). SCFAs regulate the Th2 immune response in the skin and inhibit the production of AD-related inflammatory cytokines such as IL-4, IL-13 and IL-31 to inhibit the onset of AD. Breast milk, rich in human milk oligosaccharides (HMOs), through SCFAs, IgA, and ILA help to bolster gut immunity and probiotic growth. Oral vitamin D can inhibit the colonization of *Staphylococcus aureus* (*S. aureus*) in the skin, reducing the release of virulence factors. Colonization of *S. aureus* in the skin leads to the release of virulence factors, which activate mast cell degranulation to release inflammatory cytokines IL-13. Factors such as vaginal delivery (VD) of infants and rural environments are AD -protective, whereas early antibiotic exposure disrupts probiotic colonization and elevates AD risk. Filaggrin (FLG) gene mutation is also an important risk factor for the development of AD. (Created with BioRender.com).

Neonates of mothers with GDM exhibit heightened allergen sensitivity, with susceptibility increasing by over five times. Additionally, these infants face a greater likelihood of developing atopic dermatitis, which may amplify their sensitivity risk by as much as sevenfold (Sokou et al., 2024). Additionally, the impact of maternal lifestyle during pregnancy may intervene in the offspring’s gut microbiota. A recent study collected fecal samples from infants at 3 months and 1 year of age for 16S rRNA sequence surveys and metatranscriptomics and found no significant differences in *β* diversity, the impact of GDM on the reduction of infant gut microbiota *α* diversity was only present in the early stages of life, with this effect disappearing by the time the infants were 1 year old (Chieu et al., 2024). This may be associated with the mothers in the study having undergone dietary control or insulin treatment (Figure 1).

## 4 Discussion

The gut microbiota modulates the immune response and maintains skin health through the production of metabolites such as SCFAs and indole derivatives. There is ample evidence to support the protective role of breastfeeding in establishing a diverse gut microbiota, with HMOs being indispensable in this process (Kumbhare et al., 2019; Rahman et al., 2023). Concurrently, the importance of early nutritional intervention in reducing the risk of AD should not be overlooked.

Environmental factors are another key element in shaping the gut microbiota (Yazdanbakhsh et al., 2002). The hygiene hypothesis and the potential protective effects of exposure to diverse microbial environments, such as farms, suggest that lifestyle modifications may serve as a strategy for the prevention of AD (Zhou et al., 2016).

The mode of delivery also emerged as a significant factor influencing gut microbiota colonization (Sandall et al., 2018). The relationship between CS and the risk of AD reflects that cesarean delivery may have long-term impacts on children, such as the development of eczema (Xiong et al., 2022).

The use of antibiotics, both prenatally and postnatally, is considered a disruptor of the gut microbiota and has a potential link to the development of AD (Mubanga et al., 2021). Prudent use of antibiotics is crucial for maintaining the health of the gut microbiota and may be a target for reducing the incidence of AD.

Finally, the impact of GDM on the infant gut microbiota highlights the intergenerational transmission of health risks (Soderborg et al., 2020). Maternal health care during pregnancy has a lasting impact on the offspring.

In summary, to prevent AD in children, breastfeeding, exposure to early microbial environments, vaginal delivery, reduction in antibiotic use, and the early prevention of GDM are necessary to combat the increasingly prevalent childhood AD. Future research directions could focus on the development of personalized interventions targeting the gut microbiota to effectively prevent and treat childhood AD.
